# Nanoconjugate Synthesis of *Elaeocarpus ganitrus* and the Assessment of Its Antimicrobial and Antiproliferative Properties

**DOI:** 10.3390/molecules27082442

**Published:** 2022-04-10

**Authors:** Arpitha Badarinath Mahajanakatti, Telugu Seetharam Deepak, Raghu Ram Achar, Sushma Pradeep, Shashanka K Prasad, Rajeswari Narayanappa, Deepthi Bhaskar, Sushravya Shetty, Govindappa Melappa, Lavanya Chandramouli, Sanjukta Mazumdar, Ekaterina Silina, Victor Stupin, Chandrashekar Srinivasa, Chandan Shivamallu, Shiva Prasad Kollur

**Affiliations:** 1 Department of Biotechnology, Dayananda Sagar College of Engineering, (Affiliated to VTU, Belagavi), Shavige Malleshwara Hills, Bengaluru 560078, Karnataka, India; arpitha-bt@dayanandasagar.edu (A.B.M.); rajeswari-bt@dayanandasagar.edu (R.N.); deepthibhaskar96@gmail.com (D.B.); sushravya.shetty@gmail.com (S.S.); dravidateja07@gmail.com (G.M.); lavanyac@gmail.com (L.C.); sanjukta.mazumdar25@gmail.com (S.M.); 2 Ramaiah Medical College and Hospitals, New BEL Road, Bengaluru 560054, Karnataka, India; micudeepak@yahoo.co.in; 3 Department of Biochemistry, School of Life Sciences, JSS Academy of Higher Education and Research, Mysuru 570015, Karnataka, India; rracharya@jssuni.edu.in; 4 Department of Biotechnology and Bioinformatics, School of Life Sciences, JSS Academy of Higher Education and Research, Mysuru 570015, Karnataka, India; sushmap@jssuni.edu.in (S.P.); shashankaprasad@jssuni.edu.in (S.K.P.); 5 Department of Human Pathology, Institute of Biodesign and Modeling of Complex Systems, I.M. Sechenov First Moscow State Medical University (Sechenov University), 119991 Moscow, Russia; silinaekaterina@mail.ru; 6 Department of Hospital Surgery, N.I. Pirogov Russian National Research Medical University (RNRMU), 117997 Moscow, Russia; stvictor@bk.ru; 7 Department of Biotechnology, Davangere University, Shivagangotri, Davangere 577002, Karnataka, India; 8 School of Agriculture, Geography, Environment, Ocean and Natural Sciences, Laucala Campus, The University of the South Pacific, Suva, Fiji; 9 Department of Sciences, Amrita School of Arts and Sciences, Campus, Mysuru, Amrita Vishwa Vidyapeetham, Mysuru 570026, Karnataka, India

**Keywords:** rudraksha, *Elaeocarpus ganitrus*, anticancer, antiproliferative, molecular docking

## Abstract

Cancer is one of the leading causes of death worldwide, accountable for a total of 10 million deaths in the year 2020, according to GLOBOCAN 2020. The advancements in the field of cancer research indicate the need for direction towards the development of new drug candidates that are instrumental in a tumour-specific action. The pool of natural compounds proves to be a promising avenue for the discovery of groundbreaking cancer therapeutics. *Elaeocarpus ganitrus* (Rudraksha) is known to possess antioxidant properties and after a thorough review of literature, it was speculated to possess significant biomedical potential. Green synthesis of nanoparticles is an environmentally friendly approach intended to eliminate toxic waste and reduce energy consumption. This approach was reported for the synthesis of silver nanoparticles from two different solvent extracts: aqueous and methanolic. These were characterized by biophysical and spectroscopic techniques, namely, UV-Visible Spectroscopy, FTIR, XRD, EDX, DLS, SEM, and GC-MS. The results showed that the nanoconjugates were spherical in geometry. Further, the assessment of antibacterial, antifungal, and antiproliferative activities was conducted which yielded results that were qualitatively positive at the nanoscale. The nanoconjugates were also evaluated for their anticancer properties using a standard MTT Assay. The interactions between the phytochemicals (ligands) and selected cancer receptors were also visualized in silico using the PyRx tool for molecular docking.

## 1. Introduction

Nanotechnology is the engineering and manufacturing of materials at the atomic and molecular scale. Nanotechnology refers to structures roughly in the 1–100 nm size regimes in at least one dimension [1]. It is also inherent that these materials should display properties such as electrical conductance, chemical reactivity, optical effects, etc., in a different manner when compared to bulk materials as a result of their small size. One of the important aspects in the field of nanotechnology is the development of a more consistent process for the synthesis of nanomaterials [2].

Nanomedicine is a relatively new field of science and technology. Nanomedicines can effectively interact with biological molecules compared to other therapeutics and have broadened the field of research and application. Interactions of nanodevices with biomolecules can be understood both in the extracellular medium and inside human cells [3]. Medical technologies that make use of smaller devices that are less invasive can be implanted inside the body, and, as is evidently seen, their biochemical reaction times are much shorter [4]. As compared to typical drug delivery candidates, nanodevices are faster and more sensitive [4].

Cancer is one of the diseases that is contributing to the highest number of deaths in the world. The available therapeutics, i.e., chemically synthesized drugs and some naturally derived drugs are not that effective in their tumour specific action [5]. Hence, in this regard, it can be seen that the need of the hour is to discover novel lead molecules that can be targeted against cancer cells as well as to design the appropriate drug delivery candidates to cater to the need of the specific action of the drug [5]. Nanobiotechnology, an emerging field of nanoscience, utilizes nano-based systems for various biomedical applications. This rapidly developing field of nanoscience has raised the possibility of using therapeutic nanoparticles in the diagnosis and treatment of human cancers [6]. Metal nanoparticles have a high specific surface area and surface atoms because of their outstanding physicochemical characteristics which include their optical, catalytic, electronic, magnetic, and antibacterial properties. The synthesis of metal nanoparticles is increasing to a greater extent mainly due to their potential applicability in different areas such as electronics, chemistry, energy, and medicine development [7].

Silver was known only as a metal until the recent advent of the nanotechnology domain, after which it came to be recognized as an ideal metal that could be used to produce nanoparticles. Metallic silver has been subjected to recent engineering technologies, resulting in ultrafine particles, the sizes of which are measured in nanometers (nm) and possess distinctive morphologies and characteristics [8]. Silver is well known for possessing an inhibitory result toward many bacterial strains and microorganisms commonly present in medical and industrial processes. Traditionally, nanoparticles were produced only by physical and chemical methods. Some of the commonly used physical and chemical methods are ion sputtering, solvothermal synthesis, reduction, and sol-gel technique [9]. There are two approaches for nanoparticle synthesis, namely the bottom-up and top-down approaches. In the top-down approach, scientists try to formulate nanoparticles using larger ones to direct their assembly. The bottom-up approach is a process that builds toward a larger and more complex system by starting at the molecular level and maintaining precise control of molecular structures [10]. Biosynthesis of nanoparticles is a kind of bottom-up approach where the main reaction occurring is reduction/oxidation. The need for the biosynthesis of nanoparticles rose as the physical and chemical processes were costly. Often, chemical synthesis methods lead to the presence of some toxic chemicals absorbed on surfaces that may have an adverse effect on medical applications [11]. This is not an issue when it comes to biosynthesized nanoparticles via the green synthesis route [12]. So, in the search for a cost-effective pathway for nanoparticles synthesis, scientists used microbial enzymes and plant extracts (phytochemicals). With their antioxidant or reducing properties, they are usually responsible for the reduction of metal compounds into their respective nanoparticles. Green synthesis provides advancement over chemical and physical methods as it is cost-effective, environment friendly, and easy for scaling up [13].

Rudraksha, widely acclaimed as the “King of herbal medicine”, works effectively as an antibacterial, antifungal, and anticancerous agent [14]. *Elaeocarpus sphaericus* (syn. *Elaeocarpus ganitrus*), commonly known as ‘rudraksha’ in Sanskrit and ‘rudraki’ in Hindi, is grown in the Assam and Himalayan region of India for its attractive fruit endocarps and medicinal properties. It is used in folk medicine for the treatment of stress, anxiety, depression, palpitation, nerve pain, epilepsy, migraine, lack of concentration, asthma, hypertension, arthritis, and liver diseases [15].

The present study aimed to synthesize silver nanoparticles using leaf extract of Rudraksha and characterize those using UV visible spectra, FTIR, XRD, EDX, SEM, DLS, and GC-MS. Further, the study focused on assessing these silver nanoparticles for their antibacterial, antifungal, antiproliferative, and anticancer activities.

## 2. Materials and Methods

The precursor silver nitrate was obtained from Loba chemicals (Bangalore, India). Demineralized water was collected from an ELGA RO system and was used throughout the experiments (Elga Veolia, Lane End, UK). The crystalline phases were recorded on a Bruker X-ray diffractometer with a scan range of 20–80° at a 2 °/min scan rate using Cu Kα (1.5406 Å) radiation (Bruker, Karlsruhe, Germany). The morphology and elemental composition were studied using scanning electron microscopy (SEM) and energy dispersive X-ray (EDX) mapping, respectively, which were recorded on a Zeiss microscope (Carl Zeiss, White Plains, NY, USA).

Fresh leaves of *Elaeocarpus sphaericus* were collected from Arunachal Pradesh, India in December 2017 and were authenticated at the Regional Ayurveda Research Institute for Metabolic Disorders, Bengaluru. Bacterial and fungal clinical isolates were obtained from stock cultures from the Department of Biotechnology, DSCE–Bengaluru. The MCF7- Human Breast Cancer Cell line was obtained from NCCS, Pune.

### 2.1. Processing of Samples

The fresh leaves of *Elaeocarpus sphaericus* were shade dried at room temperature for 2–4 days. The dried leaves were then hand crushed into coarse particles and were further ground using an electric grinder into finer particles and were preserved in an air-tight vessel and stored at room temperature in the dark. The powdered sample was taken for phytochemical extraction using water and methanol as the solvents. The aqueous and methanolic sample extracts prepared were of 2.5% and 5.0% concentration, respectively. The crude sample extracts were further processed by centrifuging them at 10,000 rpm for 15 min. The debris (seen as pellet) was discarded and the retained supernatant was used for the green synthesis of silver nanoparticles.

### 2.2. Phytochemical Analysis of the Crude Extract

#### 2.2.1. Gas Chromatography-Mass Spectrometry

Gas Chromatography-Mass Spectrometry (GC-MS) is one of the best-known techniques for the identification of bioactive constituents of long-chain hydrocarbons, alcohols, acids, esters, alkaloids, steroids, amino acids, and nitro compounds [16]. Thus GC-MS was used for the phytochemical analysis of the leaves of *Elaeocarpus sphaericus* for both the samples of dried powdered leaves (crude sample) and silver nanoconjugate forms using the Agilent Model 8890 GC System with Single Quadrupole Mass Spectrometer (5977B MSD) at −450 °C.

#### 2.2.2. Green Synthesis of Silver Nanoparticles

The aqueous and methanolic leaf extracts of *Elaeocarpus sphaericus* (1.25 g) were separately added to an aqueous solution of AgNO_3_ (5 mM in 20 mL distilled water) and were incubated for 24 h in the dark at room temperature for bioreduction to take place before which, they were stirred in a magnetic stirrer for 20 min [17]. After 24 h, the colloidal sample was centrifuged at 10,000 rpm for 15 min to collect the nanoconjugate in the form of a pellet. The pellets obtained from both samples (aqueous and methanolic extracts) were then washed a couple of times with distilled water and with acetone, shade dried, and stored for further experimental methods.

### 2.3. Characterization of Silver Nanoconjugate

#### 2.3.1. Ultraviolet-Visible Spectroscopy

The change in the colour of the colloidal sample after 24 h indicates the formation of silver nanoconjugate by bioreduction and the simplest way to confirm this theory is by the use of UV-Vis spectroscopy which works on the principle of Surface Plasmon Resonance (SPR) absorption bands for silver nanoparticles characterization [18]. The absorbance spectrum of the colloidal samples was examined in the range of 400–500 nm, using a UV-Vis spectrometer double beam UV-1700 Series with distilled water and methanol as the blank for aqueous and methanolic colloidal samples (silver nanoconjugate), respectively.

#### 2.3.2. Fourier-Transform Infrared Spectroscopy

The FTIR characterization technique was conducted for the nanoconjugates obtained from both the aqueous and methanolic extract of the samples with Spectrum Two PIKE instrumentation with a scanning range of 4000–6000 cm^−1^ to classify the functional groups involved in the biotagging and in turn, to predict the phytochemicals present on the silver nanoconjugate.

#### 2.3.3. X-ray Diffraction Analysis

Being a non-destructive technique, X-ray diffraction analysis was carried out to obtain information about the structural attributes (presence of silver at a nanoscale) of the nanoparticles. XRD patterns were recorded in the system operating at a voltage of 40 kV and a current of 30 mA with 3CuK α radiation (λ = 1.54060/1.54443 Å) and the diffracted intensities were recorded from 0° to 70° 2θ angles.

#### 2.3.4. Energy-Dispersive X-ray Spectroscopy

Employment of energy-dispersive X-ray spectroscopy was to affirm the presence of silver in the sample and also to find the other elementary compositions in the sample as the EDX assay is used for elemental assay or chemical characterization of a sample [19].

#### 2.3.5. Dynamic Light Scattering

DLS Particle Size and Zeta Potential Analyzer is a commonly used system for nanoparticles, colloidal, and macromolecular characterization [20] and thus was utilized for obtaining the particle size distribution profile and zeta potential of the silver nanoconjugate. For molecules and particles that are small enough, a highly negative zeta potential will confer stability.

#### 2.3.6. Scanning Electron Microscope

For determining the surface morphology and size of the silver nanoconjugate, electron microscope scanning was conducted [21] using the SEM S3400.

### 2.4. Assessment of Antibacterial Activity

#### 2.4.1. Disc Diffusion Assay

A standard disc diffusion assay was carried out to assess the antibacterial activity of the silver nanoconjugate using clinical isolates of *Escherichia coli*, *Klebsiella pneumoniae*, *Pseudomonas aeruginosa*, *Staphylococcus aureus*, and *Bacillus cereus*. Whatman filter discs saturated with samples of four different concentrations of 100% (1mg/mL), 75%, 50%, and 25% were placed on the bacterial lawn cultured Petri plate after air-drying, along with distilled water as control and Chloramphenicol as standard. After 48 h of incubation at 37 °C, the zone of inhibition was measured by taking diameters on four different sides around the radial zone observed.

#### 2.4.2. Biofilm Assay

Seed inoculum of *Bacillus cereus* was added into four clean test tubes having 10 mL of nutrient broth and 1 mL of the sample with four different concentrations of 100% (1 mg/mL), 75%, 50%, and 25% except for the control which had 1 mL of distilled water. The OD reading of the incubated cultures was taken at 610 nm after 24 h incubation at 37 °C. A volume of 2 mL of the samples from each test tube was kept aside for further use and then a single drop of crystal violet was added to all test tubes to observe staining colour gradation. To confirm whether the OD reading obtained was due to biofilm or cell death, we reinoculated the samples by taking 25 mL of nutrient broth and 500 μL of incubated samples that were tested for the OD reading. These reinoculated samples were incubated at 37 °C and were also subjected to growth pattern monitoring by taking an OD reading every one hour at 610 nm to observe the effect of the test extract on the bacterial culture. Finally, the colony viability in the reinoculated sample was verified using the pour plate technique.

#### 2.4.3. Assessment of Antifungal Activity

Antifungal activity of the synthesized silver nanoconjugate was determined qualitatively using a standard disc diffusion assay on clinical isolates of *Penicillium notatum*, *Aspergillus flavus*, *Aspergillus niger*, *Trichothecium roseum*, *Fusarium oxysporum*, and *Trichoderma harzianum*. On the lawn cultured fungal plates, Whatman filter discs saturated with samples of four different concentrations of 100% (1mg/mL), 75%, 50%, and 25% were placed after air-drying along with distilled water as control. After 48–62 h of incubation at room temperature, the zone of inhibition was measured by taking the diameter on four different sides around the radial zone observed.

#### 2.4.4. Evaluation of Antiproliferative Activity

Anticancer drug discovery involves the identification of compounds that kill or inhibit the growth of cancer cells. These involve the use of various models to screen for the presence of cytotoxic activities or antiproliferative activities. Among the various model systems, the most cost-effective model for evaluating cytotoxicity is the yeast (*Saccharomyces cerevisiae*) model [22]. A volume of 0.5 mL of yeast seed inoculum, 2.5 mL Potato Dextrose Broth, and 1 mL of the sample of four concentrations that are 100% (1 mg/mL), 75%, 50%, and 25% was added with distilled water as control. After 24 h incubation at 37 °C, the OD of incubated cultures was taken at 610 nm. The sample cultures were smeared and stained with 0.125% methylene blue then the slides prepared were observed under a light microscope at 40× and 100× magnification to visualize the effect of the test extract on the cellular integrity.

### 2.5. Anticancer Activity

#### MTT Assay

The MTT assay is a colorimetric assay used for the determination of cell proliferation and cytotoxicity. A standard MTT assay was conducted using the breast cancer cell line MCF-7. Seeding of 200 μL of cell suspension in a 96-well plate at the required cell density (20,000 cells per well), without the test agent (silver nanoconjugates), was performed and the cells were allowed to grow to attain confluency. The cells were treated with varying concentrations (25 μg, 50 μg, 100 μg, 200 μg, and 400 μg) of the test agents and incubated for 24 h at 37 °C in a 5% CO_2_ atmosphere. After the incubation period, the spent media was removed and the MTT reagent was added to a final concentration of 0.5 mg/mL of total volume. The plates were returned to the incubator for 3 h. Then, the MTT reagent was washed and 100 μL of solubilization solution (DMSO) was added [23]. The absorbance was read at 570 nm with DMSO as a blank. The IC_50_ value was determined by using a linear regression equation, i.e., Y = Mx + C. Here, Y = 50, M and C values were derived from the viability graph.

### 2.6. Prediction of Interaction of Phytochemicals against Selected Cancer Receptors

The underlying aim of molecular docking is to predict the ligand-receptor complex structure using computational methods and PyRx software is one of the user-friendly ways of Generic Evolutionary Method for molecular docking [24]. BRCA1 and CCNDI receptors were chosen from a literature survey and their structures were downloaded from the Protein Data Bank with PDB ID 4OFB and 2W9F, respectively [25]. The downloaded protein structures were further edited accordingly to delete the non-standard residues and water molecules attached. The receptors chosen for the study play a significant role in the breast cancer pathway [26]. The hydrophobic pocket-forming amino acid residues where the ligand binds to the protein structures were obtained using CASTp online tool [27].

The ligands were selected from a list of active compounds obtained from GC-MS results after ADMET testing and were named as lig1(Beclomethasone), lig2 (18, 19-Seco-15á- yohimban-19-oic acid, 20, 21-didehydro-16á-(hydroxymethyl)-, methyl ester), lig3 (Benzimidazole, 2-(4-nitrophenyl)-5-(thien-2-ylcarbonyl)-1-hydroxy-3-oxide), lig4 (Strychnidin-10-one,2,3-dimethoxy-19-oxide), and lig5 (Benzothiophene-3-carboxylic acid,4,5,6,7-tetrahydro-6-tert-butyl-2-cyclopropanoyl-amino-ethyl ester). All five ligands structures were sketched using Chemsketch [28] and were converted to the required pdb format file using OpenBabel software [29]. Finally, the structures of the ready ligands were docked with receptors using PyRx [30].

## 3. Results and Discussion

### 3.1. Processing of Samples

Drying of the leaves rendered them to be more brittle which aided in better grinding to finally obtain a finely powdered form of the leaves that would in turn effectively contribute to the excessive extraction of the active phytochemicals present. Solvent extracts were prepared from the powder using two different types of solvents: water (a polar inorganic solvent) and methanol (a non-polar organic solvent).

### 3.2. Phytochemical Analysis of the Crude Extract

**Figure 1:**
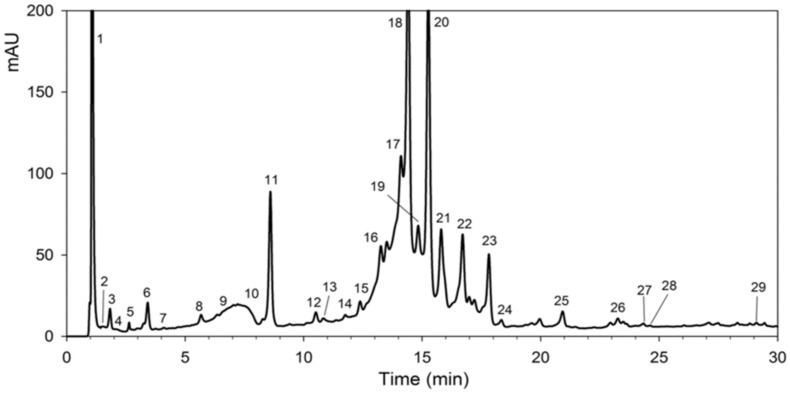
Chromatogram and identified compounds of the crude leaf extract obtained from GC-MS.

The GC-MS technique aided in confirming the presence of compounds in the extract based on the prominent peaks displayed as depicted in Figure 1. The GC-MS analysis of the extract showed five different phytochemicals and they were identified based on retention time and chromatograms by comparing standards. The quantitative analysis for phytochemicals was made based on GC-MS results and was made based on the height of the peaks of each compound obtained in GC-MS. Each compound’s height clearly indicates the quantity of each compound present in the extract.

### 3.3. Green Synthesis of Silver Nanoparticles

A significant colour change from pale yellow to gray was visually assessed after 24 h of incubation in the dark and this was attributed to the bioreduction phenomenon that had occurred in the presence of the nitrate radical and the phytochemicals. This clearly indicated the formation of nanoparticles as the significant change in the physical attributes of the particles, i.e., the change in the set of wavelengths it absorbed or scattered before and after incubation itself validates the phenomenon of bioreduction.

### 3.4. Characterisation of Silver Nanoconjugate

#### 3.4.1. Ultraviolet-Visible Spectroscopy

**Figure 2:**
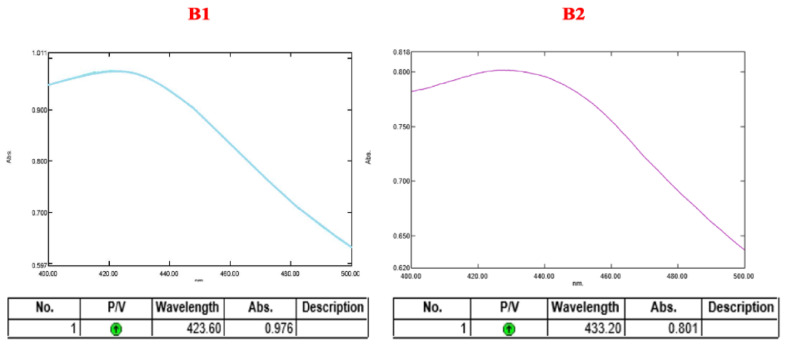
UV-Vis Spectra of nanoconjugates formed from (**B1**) aqueous extract and (**B2**) methanolic extract.

Metal nanoparticles are known to exhibit a distinct Surface Plasmon Resonance (SPR) absorption band which results from a mutual interaction of free electrons of the metal nanoparticles and the light wave which are vibrating in resonance. The characteristic SPR peak for metallic silver is usually seen at 424 nm [31]. However, for the colloidal silver nanoparticles, the absorption band would lie in the range of 400–450 nm [32]. The UV peaks were obtained at 423.60 nm and 433.20 nm for the nanoconjugates formed from aqueous and methanolic extracts, respectively, as depicted in Figure 2.

#### 3.4.2. Fourier Transform Infrared Spectroscopy

**Figure 3:**
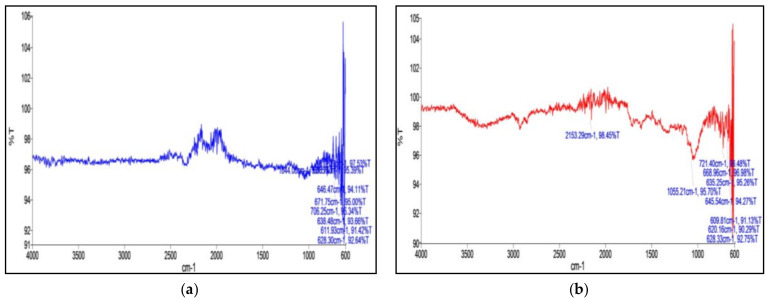
FTIR Spectra of nanoconjugates formed from (**a**) aqueous extract and (**b**) methanolic extract.

From the spectra obtained, characteristic wave numbers aided in the identification of the probable bonds or functional groups involved in the tagging of phytochemicals were deduced as follows (Figure 3). A clear occurrence of a polar C-N bond in the aqueous extract indicated the difference between the two different types of nanoconjugates synthesized.

#### 3.4.3. X-Ray Diffraction Analysis

**Figure 4:**
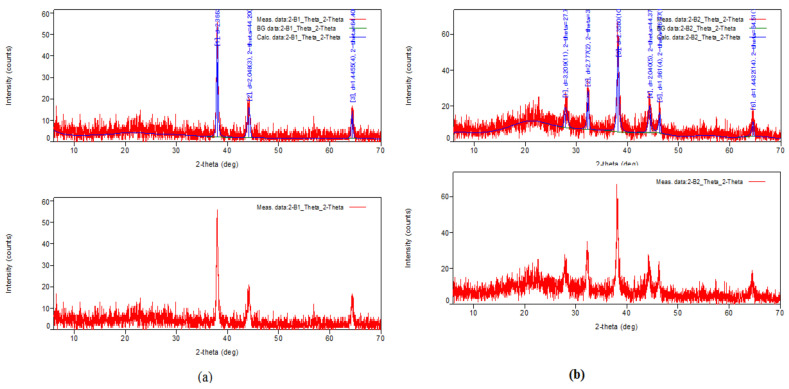
XRD profile of nanoconjugates formed from (**a**) aqueous extract and (**b**) methanolic extract.

The XRD profile of the nanoconjugates yielded characteristic peaks for a silver nanoparticle at a 2θ value of 38°, 44°, and 64° (after comparison with the JCPDS file no 89-3722) which validated the presence of silver nanoparticles in the test samples (Figure 4). These coordinates of 2θ directly correspond to the planes (111), (200), and (220). This pattern is found to be characteristic of the face-centered cubic lattice structure. Similar results have been reported in earlier works [33,34]. The other weak peaks observed are due to the phytochemicals present in the leaf extract.

#### 3.4.4. Energy Dispersive X-Ray Spectroscopy

**Figure 5:**
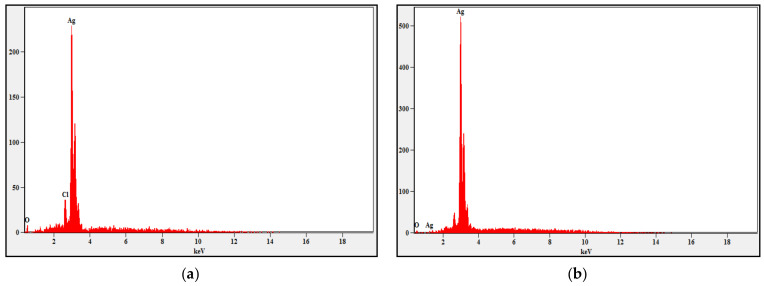
EDAX Spectrum of nanoconjugates formed from (**a**) aqueous extract and (**b**) methanolic extract.

From the spectra, important deductions about the elemental composition were made. A prominent peak of silver confirmed the presence of silver as a constituent in the sample. Moreover, a characteristic peak at 3keV for metallic silver further validated the phenomenon of SPR [28]. The Presence of the ‘Cl’ atom was very evidently highlighted in the nanoconjugate sample prepared from the aqueous extract (Figure 5).

#### 3.4.5. Dynamic Light Scattering

**Figure 6:**
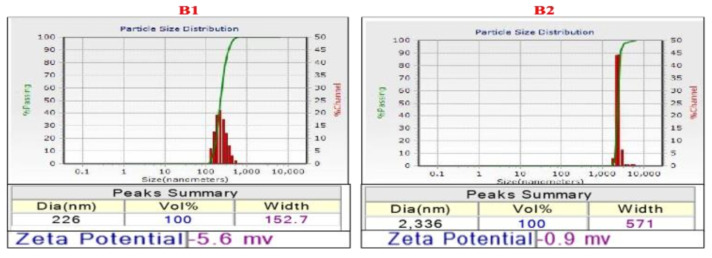
DLS size distribution graph of nanoconjugates formed from (**B1**) aqueous extract and (**B2**) methanolic extract.

The particle size distribution graph was key in analyzing the mean diameter of the sample. It was observed that the nanoconjugates synthesized from the aqueous extract were much smaller in size compared to the other type. The mean diameters of nanoconjugates made from aqueous and methanolic extract were 226 nm and 2,336 nm, respectively (Figure 6). This difference could be attributed to the phytochemicals tagged and also the extent of stearic hindrance experienced amongst the phytochemicals. Furthermore, the zeta potential of the aqueous extract nanoconjugates was more negative which accounted for their stability. In this view, they were considered to be more feasible candidates for further experimental analysis or applications.

#### 3.4.6. Scanning Electron Microscopy

**Figure 7:**
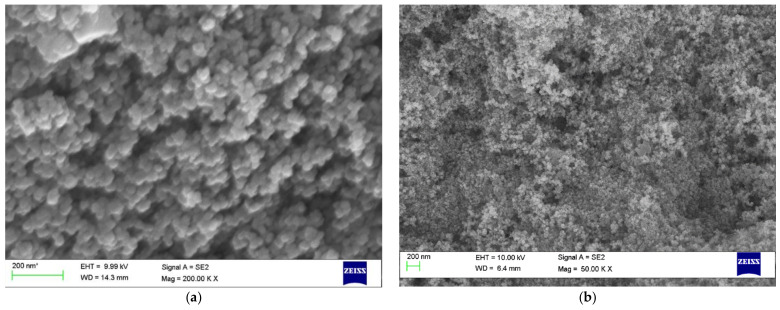
SEM images of the nanoconjugates formed from (**a**) aqueous extract and (**b**) methanolic extract.

SEM analysis rendered secondary electron images of the sample (Figure 7) from which it was very much evident that the nanoconjugates were mostly spherical in geometry with a smooth surface morphology in both cases.

### 3.5. Assessment of Antibacterial Activity

#### 3.5.1. Agar Disc Diffusion Assay

**Figure 8:**
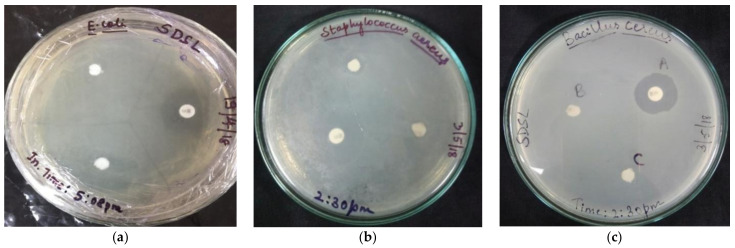
Disc diffusion assay plates displaying the zone of inhibition for (**a**) *Escherichia coli* (**b**) *Staphylococcus aureus* and (**c**) *Bacillus cereus*.

A zone of inhibition was observed for all the different bacterial strains used. Different bacterial strains were inhibited at different concentrations of the nanoconjugate extract. Among the four different concentrations used, a concentration of 0.5 mg/mL was seen to be effective against all the strains to various degrees (Figure 8).

#### 3.5.2. Biofilm Assay

**Figure 9:**
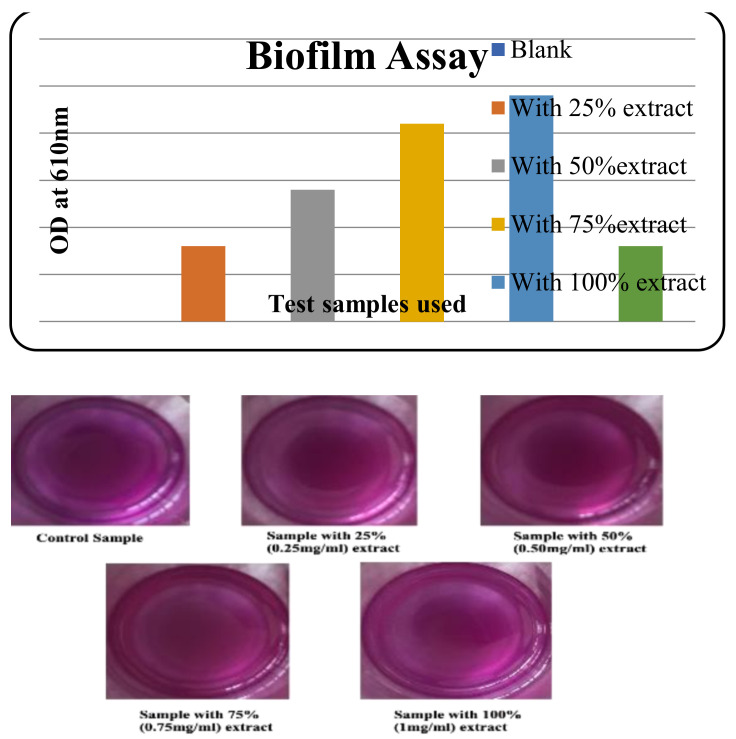
Biofilm assay of as-prepared silver nanoconjugates.

**Figure 10:**
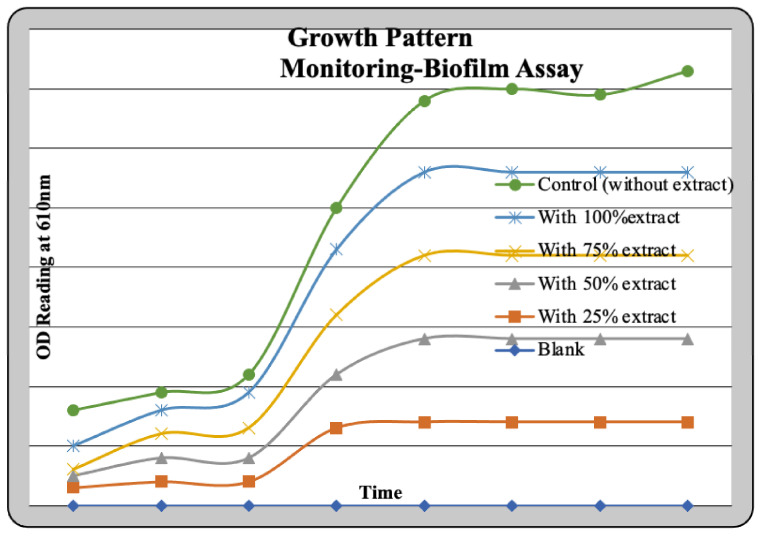
Growth pattern monitoring-biofilm assay.

Optical density measurements after a time period of 24 h were taken to test for the action of the nanoconjugate extract. The same samples were also subjected to a staining reaction with Crystal Violet. The result obtained was as depicted in the graph (Figure 9). To justify that the OD readings were due to the process of cell lysis, a reinoculation was carried out to test for the viability of the cells. This was undertaken to monitor the growth pattern of the culture and the action of the test sample in parallel. The growth pattern curve for different test and standard samples can be graphically represented (Figure 10). A considerable degree of growth was seen in the control which clearly justifies the presence of viable cells in the control sample. On the other hand, test samples had shown stagnation in growth. This result was further validated using a pour-plate technique.

#### 3.5.3. Assessment of Antifungal Activity

**Figure 11:**
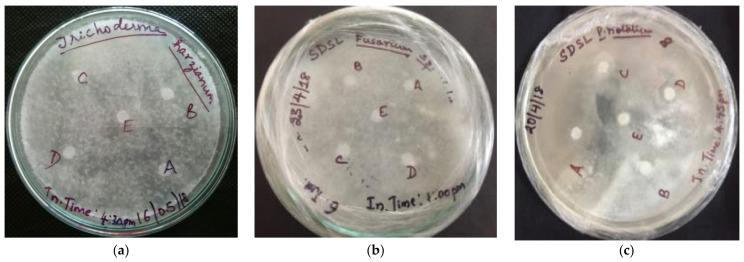
Disc diffusion assay testing for the antifungal activity (**a**) *Trichoderma harzianum* (**b**) *Fusarium oxysporum* and (**c**) *Penicillium notatum*.

A standard agar disc diffusion assay was carried out and it was observed that *Aspergillus niger*, *Aspergillus flavus*, *Penicillium notatum*, and *Trichothecium roseum* exhibited a clear zone of growth inhibition due to the action of the nanoconjugated extract at different working concentrations. Two fungal strains, *Fusarium oxysporum* and *Trichoderma harzianum*, did not show any degree of growth inhibition (Figure 11) for the four different concentrations of the extract used (stock being 1 mg/mL).

#### 3.5.4. Evaluation of Antiproliferative Activity

**Figure 12:**
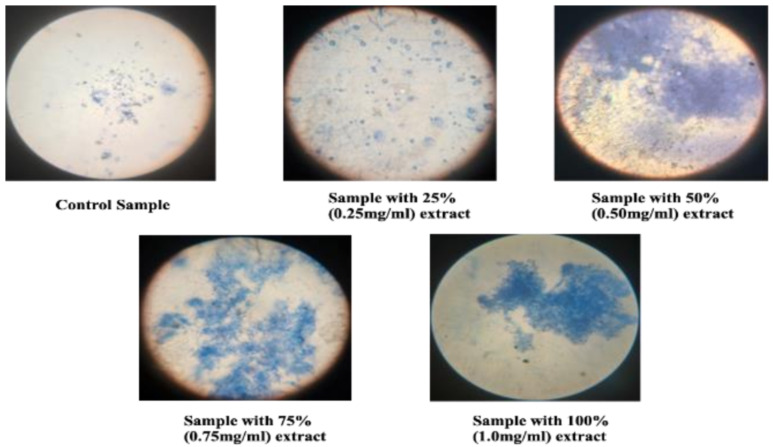
Antiproliferative activity as observed in the microscope (after staining with methylene blue).

From the microscopic field views of the test and control samples displayed, it can be noted that the control sample, which was devoid of the extract, contained metabolically active cells. Moreover, it was also observed that these cells continued to be actively involved in the process of budding. On the other hand, in the test samples that contained different concentrations of the extract, the budding process was obstructed. Further, in the test samples with a higher concentration of the extract, an extensively higher degree of cell lysis was clearly visualized. The extent of cell burst was quantified by the OD measurements taken at 610 nm which directly signified the amount of cell debris released into the sample (Figure 12).

### 3.6. Assessment of Anticancer Activity

#### MTT Assay

**Figure 13:**
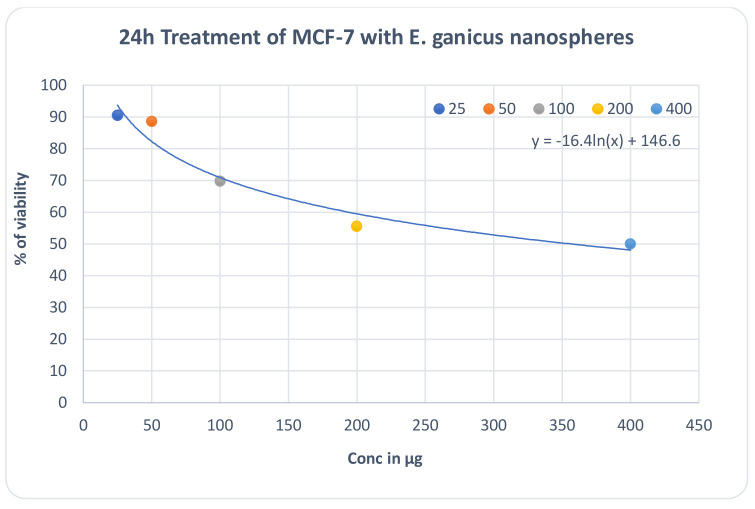
Graphical representation of the MTT assay-cell viability test results.

**Figure 14:**
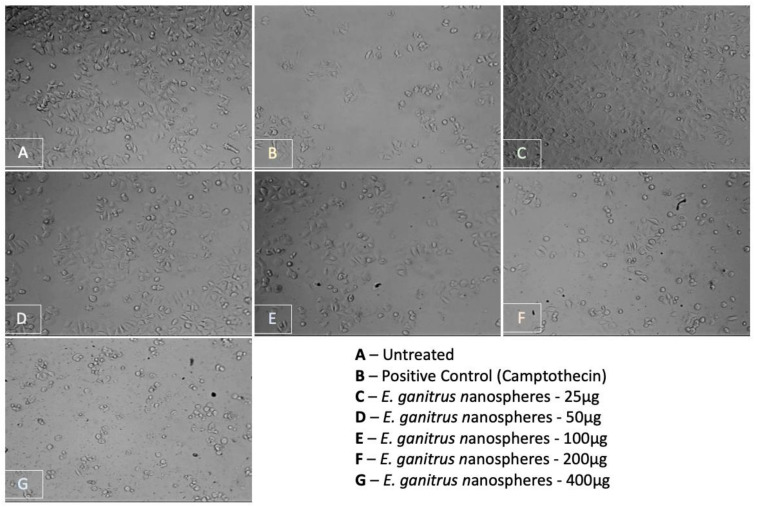
Photomicrographs of MCF-7 cells treated for 24 h with EGNs.

Potential dose-dependent anticancer activity was observed upon treatment of MCF-7 cells with the test sample, i.e., the nanoconjugates synthesized from the aqueous extract. Camptothecin was used as a positive control in the assay and it was observed that the test extract reflected a similar effect on the cell line as that of the standard drug (0.5 µM) at its highest treatment concentration of 400 µg. From the graphical representation of the result, the IC_50_ value of the test extract was deduced to be 361.49 µg (Figure 13). The photomicrographs of the cells treated with the test sample indicate a change in cellular morphology upon treatment, suggesting the efficacy of the treatment (Figure 14) (Prasad SK, Veeresh PM, Ramesh PS, Natraj SM, Madhunapantula SV, Devegowda D). Phytochemical fractions from *Annona muricata* seeds and fruit pulp inhibited the growth of breast cancer cells through cell cycle arrest at G_0_/G_1_ phase. Furthermore, the cellular population has evidently decreased conversely to the increase in treatment concentration.

### 3.7. Prediction of Interaction of Phytochemicals against Selected Cancer Receptors-Molecular Docking

**Figure 15:**
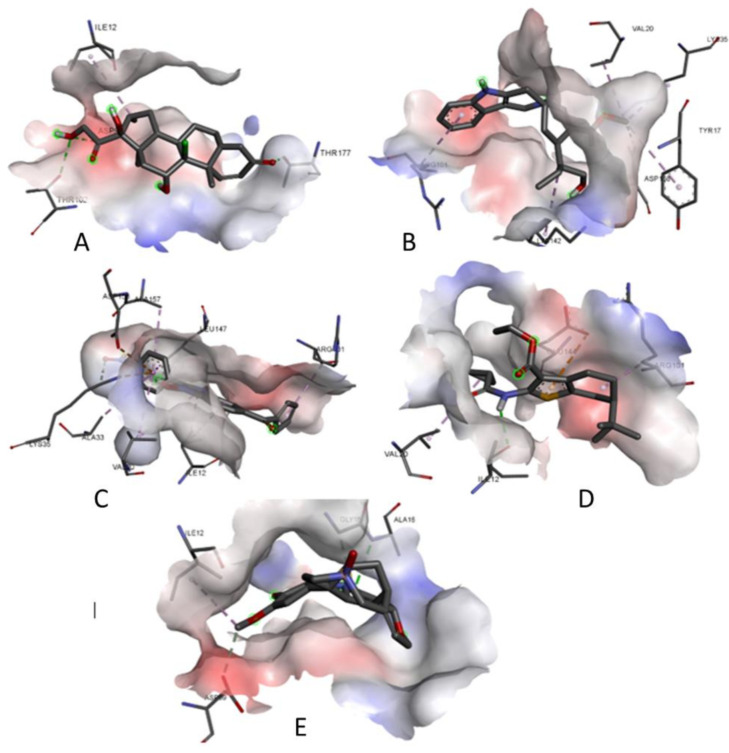
Molecular Docking Results of (**A**) lig1, (**B**) lig2, (**C**) lig3, (**D**) lig4 and (**E**) lig5 with CCND1 cancer receptor (2WF9).

**Figure 16:**
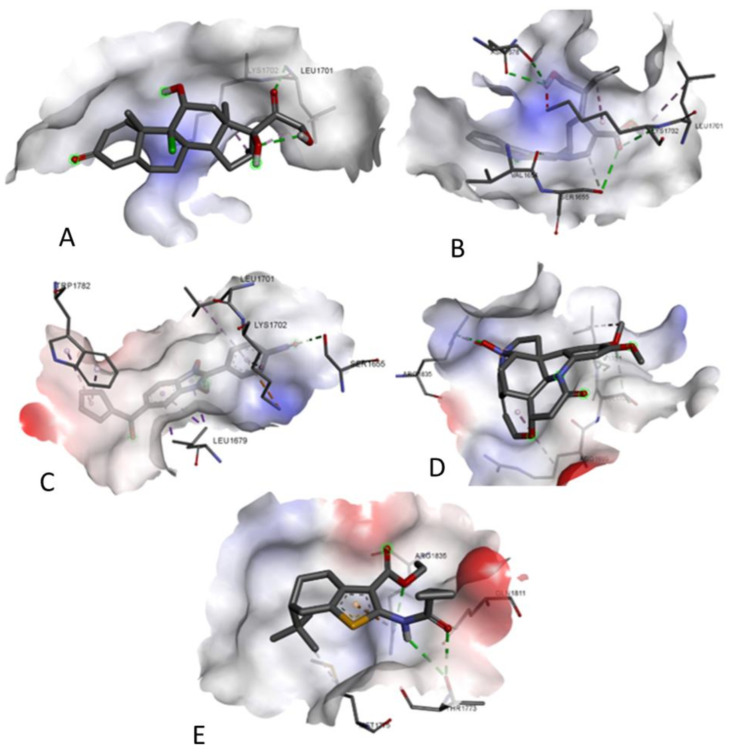
Molecular Docking Results of (**A**) lig1, (**B**) lig2, (**C**) lig3, (**D**) lig4 and (**E**) lig5 with BRCA1 cancer receptor (4OFB).

Figure 15 shows the Molecular Docking Results of ligands with the CCND1 cancer receptor (2WF9). After docking the ligands against the selected cancer receptors, a considerably good degree of interaction was observed [35]. Amongst the two receptors used for molecular docking, the best results were obtained for the BRCA1 gene (PDB ID: 4OFB) with binding energy ranging from −5.6 kJ/mol to −7.7 kJ/mol. The best-docked poses of the ligands with the receptors were analyzed using the BIOVIA Discovery Studio visualizer and are depicted in the figure (Figure 15 and Figure 16) [36]. The binding energy and interacting residues are represented in Table 1.

## 4. Conclusions

The synthesized silver nanoconjugates of the leaf extracts of *Elaeocarpus sphearicus* were found to be physically stable and pharmacologically active. Between the two types of nanoconjugates prepared from the aqueous and methanolic leaf extracts by the method of green synthesis, the nanoconjugates formulated from the aqueous leaf extract were found to be more stable as they were feasible both in terms of size and zeta potential.

The nanoconjugates exhibited significant antibacterial activity against five clinical isolates, namely, *Escherichia coli*, *Klebsiella pneumonia*, *Pseudomonas aeroginosa*, *Staphylococcus aureus*, and *Bacillus cereus* along with efficient antifungal properties for the selected fungal strains, *Aspergillus niger*, *Aspergillus flavus*, *Penicillium notatum*, and *Trichothecium roseum* at the four different working concentrations of the nanoconjugates. The results from antiproliferative testing portrayed that the nanoconjugates potentially affect the cell division process and viability of fast-growing cells such as *Saccharomyces cerevisiae* (yeast) at as low a concentration as 0.50 mg/mL of the sample.

Based on the MTT assay result, it can be summarized that the nanoconjugates possessed potential anticancer activity and the IC_50_ value was found to be 361.49 µg against the MCF-7 breast cancer cell line, comparable to the Campothecin positive control. The need now is to validate the observed cytotoxicity in vivo and to elucidate the mechanistic basis for the same. Furthermore, molecular docking performed using PyRx demonstrated the favourable set of interactions for the selected cancer receptors (based on the cell line) and phytochemicals of *Eleaocarpus sphearicus* identified from a GC-MS analysis.

## Figures and Tables

**Table 1:** Molecular Docking binding energy and interaction details.

Receptor ID	Ligand Names	Binding Energy (kJ/moL)	Interacting Residues
2WF9	Lig1	−5.6	ILE-12, ASP-99, THR-102, THR-177
	Lig2	−7.3	TYR-17, VAL-20, LYS-35, ARG-101, LYS-142, ASP-158
	Lig3	−8.1	ILE-12, VAL-20, ALA-33, LYS-35, ARG-101, LEU-147, ALA-157, ASP-158
	Lig4	−7.0	ILE-12, VAL-20, ARG-101, GLU-144
	Lig5	−5.4	ILE-12, GLY-15, ALA-16, ASP-99
4OFB	Lig1	−6.2	GLY-1656, LEU-1701, LYS-1702, ASN-1774
	Lig2	−6.0	VAL-1654, SER-1655, ASN-1678, LEU-1701, LYS-1702
	Lig3	−7.7	SER-1655, LEU-1679, LEU-1701, LYS-1702, TRP-1782
	Lig4	−6.3	ARG-1699, THR-1700, LEU-1701, LEU-1839
	Lig5	−5.6	THR-1773, MET-1775, GLN-1811, ARG-1835

## Data Availability

Not Applicable.

## Sample Availability

Samples of the compounds are available from the authors.
